# Crosstalk of the Wnt/β-Catenin Signaling Pathway in the Induction of Apoptosis on Cancer Cells

**DOI:** 10.3390/ph14090871

**Published:** 2021-08-28

**Authors:** Cristina Trejo-Solis, Angel Escamilla-Ramirez, Dolores Jimenez-Farfan, Rosa Angelica Castillo-Rodriguez, Athenea Flores-Najera, Arturo Cruz-Salgado

**Affiliations:** 1 Laboratorio Experimental de Enfermedades Neurodegenerativas, Instituto Nacional de Neurología y Neurocirugía, Ciudad de Mexico 14269, Mexico; eramed118@gmail.com (A.E.-R.); tlacaelel333@gmail.com (A.C.-S.); 2 Laboratorio de Inmunología, División de Estudios de Posgrado e Investigación, Facultad de Odontología, Universidad Nacional Autónoma de México, Ciudad de Mexico 04510, Mexico; farfanmd@unam.mx; 3 CONACYT-Instituto Nacional de Pediatría, Ciudad de Mexico 04530, Mexico; racastilloro@conacyt.mx; 4 Centro Médico Nacional 20 de Noviembre, Departamento de Cirugía General, Ciudad de Mexico 03229, Mexico; atheneafloresnajera@gmail.com

**Keywords:** crosstalk, β-catenin, apoptosis, signaling, cancer cells

## Abstract

The Wnt/β-catenin signaling pathway plays a major role in cell survival and proliferation, as well as in angiogenesis, migration, invasion, metastasis, and stem cell renewal in various cancer types. However, the modulation (either up- or downregulation) of this pathway can inhibit cell proliferation and apoptosis both through β-catenin-dependent and independent mechanisms, and by crosstalk with other signaling pathways in a wide range of malignant tumors. Existing studies have reported conflicting results, indicating that the Wnt signaling can have both oncogenic and tumor-suppressing roles, depending on the cellular context. This review summarizes the available information on the role of the Wnt/β-catenin pathway and its crosstalk with other signaling pathways in apoptosis induction in cancer cells and presents a modified dual-signal model for the function of β-catenin. Understanding the proapoptotic mechanisms induced by the Wnt/β-catenin pathway could open new therapeutic opportunities.

## 1. Introduction

The Wnt/β-catenin pathway is severely dysregulated in various cancer cell types [1,2,3]; its components participate in the regulation of cell survival, proliferation, epithelial-mesenchymal transition, inflammation, angiogenesis, migration, invasion, and stem cell renewal [4,5,6]. Furthermore, it has been demonstrated that the Wnt/β-catenin pathway induces tumor-suppressive effects on several cancer cells [7,8,9,10]. The role of β-catenin expression in cancer is debated, as it has been found to be involved both in pro- and antitumoral functions. It was already known that the Wnt pathway could be associated with both a negative and positive patient outcome in several tumor types [11].

This dichotomic role of β-catenin can be explained considering that certain proteins show both oncogenic and suppressive actions on a subset of cancer cells [8,12,13,14]. On the other hand, the crosstalk of signaling pathways can extend the functions of individual pathways and result in a more complex regulatory network, inherent to the diversity and homeostasis of biological systems [12]. The overexpression of β-catenin in lung cancer cells does not induce lung tumors in mice, while the co-expression of β-catenin and the KRAS onco-gene results in lung and melanoma tumors [15,16].

On a different note, it has been demonstrated that both overactivation and inhibition of the Wnt/β-catenin pathway induce antineoplastic effects even in the same cancer type [11,17]; thus, an imbalance in the threshold of this pathway could open promising therapeutic strategies [17,18]. Duffy et al. showed that either hyperactivating or inhibiting the Wnt/β-catenin signaling may lower cell viability rates, via apoptosis induction when the pathway is overactivated, or by inducing cell cycle arrest and differentiation when it is inhibited in malignant melanoma, colon carcinoma, and neuroblastoma tumors [17]. Those authors suggest that the Wnt/β-catenin pathway shows a bidirectional vulnerability, which could open new therapeutic approaches for these and other tumor types [17]. Furthermore, it has been reported that the Wnt/β-catenin signaling could either induce or block tumor initiation and promotion, as well as invasion, metastasis, and drug resistance in a tumor-stage- and tumor-type specific manner [11].

A correlation between the activation of the Wnt/β-catenin pathway and the induction of apoptosis via extrinsic and intrinsic pathways, involving the overexpression of c-myc, cyclin D, and Bcl-2 Associated X (Bax), the activation of the BH3-interacting domain death agonist (Bid), caspases-8, -9, and -3, and a downregulation of B-cell lymphoma 2 (Bcl-2) and Sox-1 has also been observed [19,20,21]. Those authors suggested that a downregulation of Sox-1 promotes the activation of β-catenin, which can induce the overexpression of cyclin D and c-myc, which leads to apoptotic cell death [19,20,21,22]. It was found that Sox-1 binds β-catenin, favoring the inactivation of the Wnt/β-catenin pathway and decreasing the expression of c-myc and cyclin D [23]. On the other hand, it has been demonstrated that c-myc and cyclin D induce apoptosis under different stimuli [22]. Decreased E-cadherin levels induce the nuclear accumulation of β-catenin, which promote the stability of p53 and provide a selective pressure for the loss of wild-type p53 function in cancer cells [24,25]. It has been suggested that the Wnt/β-catenin pathway can induce an overexpression of c-myc, which increases the levels of p14^ARF^ (a Mdm2 inhibitor), promoting the nuclear accumulation of p53 [24].

Altogether, these results suggest that the initial assumption that the overexpression of the Wnt/β-catenin pathway promoted the initiation, promotion, and progression of cancer and led to a worse clinical outcome could be an oversimplification [11]. Instead, it seems that the Wnt pathway, either β-catenin-dependent or independent, could induce or inhibit carcinogenesis in a context-dependent manner. In this review, we summarize current evidence indicating that signaling networks related to Wnt/β-catenin induce antineoplastic effects in cancer cells and discuss the underlying molecular mechanisms. We start by introducing the Wnt/β-catenin pathway, the basic machinery of apoptosis, and the findings of various studies that demonstrated the proapoptotic effect of β-catenin in cancer models. Our goal is to provide a more nuanced picture, portraying the complex interactions of this pathway in various cellular contexts, and to highlight that its activation does not always result in cancer progression. This knowledge may help us to develop more effective therapies.

## 2. The Wnt Pathways

Wnt proteins can activate at least three different signaling pathways, known as the canonical or β-catenin pathway, and two β-catenin-independent, non-canonical pathways, known as the Wnt/Ca^2+^ or calcium pathway, and the Wnt/planar cell polarity (PCP) pathway [26].

### 2.1. Wnt/Planar Cell Polarity (PCP) Pathway

**Figure 1:**
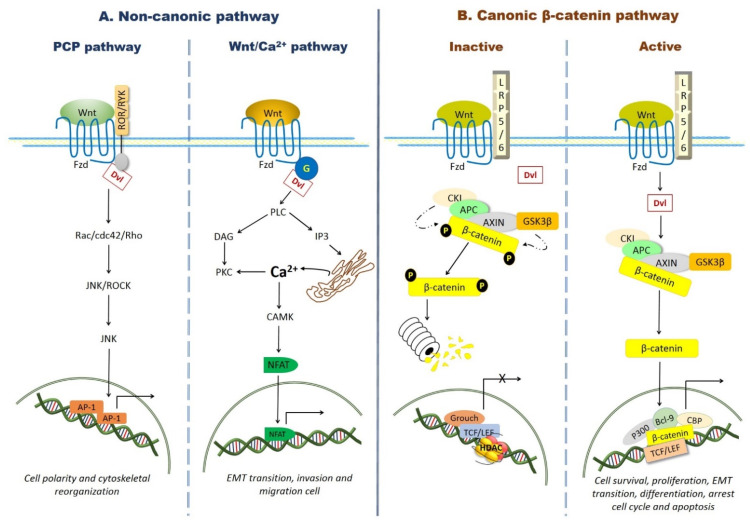
Non-canonical and canonical Wnt pathways. In the non-canonical Wnt/PCP and Wnt/Ca^2+^ pathway (**A**), the interaction of Wnt/Fzd/ROR and RYK promotes the activation of small GTPases like Rac1, Cdc42, and RhoA, which activate JNK and ROCK, inducing cell polarity and cytoskeletal reorganization through the expression of the target gene induced by the AP-1 transcriptional factor (PCP pathway). The interaction of Wnt/Fzd with G proteins induces the activation of PLC, promoting the cleavage of PIP_2_ to DAG and PIP_3_, causing the activation of PKC and calcium release by IP_3_ from the endoplasmic reticulum. Calcium activates CAMK, with the subsequent transactivation of NFAT and the regulation of EMT, cell invasion, and migration (Wnt/Ca^2+^ pathway). With respect to the canonical Wnt/β-catenin pathway (**B**), in the absence of Wnt ligands, the pathway is turned off. Cytosolic β-Catenin is phosphorylated by a Destruction Complex formed by APC, Axin, CKI, and GSK3β, which lead to its proteasomal degradation. In the nucleus, the transcriptional factors TCF/LEF bind the transcriptional repressors Groucho/HDAC. On the other hand, when activated, Wnt binds Fzd/ Lrp5/6, activating Dvl, which recruits Axin, favoring the dissociation and inactivation of the Destruction Complex and an ensuing cytoplasmic accumulation (and a nuclear accumulation at latter times) of β-catenin; in the nucleus, β-catenin interacts with TCF/LEF and transcriptional coactivators like Bcl-9, CBP, and p300 to transcribe its target genes, regulating cell survival, proliferation, EMT transition, differentiation, cell cycle arrest, and apoptosis. Continue arrows (↓) indicate activation; arrows with (⊥) indicate inhibition.

The Wnt/PCP pathway regulates cell migration, synaptogenesis, axonal growth/guidance, and ciliogenesis. Wnt proteins such as Wnt 5a and Wnt 11 interact with the FZ receptors and with the ROR and RYK coreceptors, activating small guanosine triphosphatases (GTPases) like the RAS-related C3 botulinum toxin substrate 1 (Rac1), Cdc42, and Rho A, which in turn activate the c-Jun N-terminal kinase (JNK) pathway and the Rho-associated kinase (ROCK), leading to gene expression and cytoskeleton remodeling [27,28] (Figure 1).

### 2.2. Wnt/Ca^2+^ Pathway

This pathway is involved in the induction of the epithelial–mesenchymal transition, as well as in cell migration and invasion [29]. The pathway is activated when Wnt proteins (Wnt 5a and Wnt 11) bind the Fz receptor, triggering the activation of the proteins G and phospholipase C (PLC) and leading to an increased release of Ca^+2^ from mitochondria and the endoplasmic reticulum, which in turn activates calcium-dependent proteins like the calcium calmodulin mediated kinase II (CAMKII) and the protein kinase C (PKC), both of which induce the activation of the transcriptional factor NFAT [30] (Figure 1).

### 2.3. β-Catenin Canonical Pathway

The main mediator of this signaling pathway is the cytoplasmic protein β-catenin, which acts as a molecular hub for multiple signals and regulates various cell functions. In cell adhesion, β-catenin binds E-cadherin in the plasma membrane and α-catenin in the cytoplasm. E-cadherin stabilizes β-catenin through α-catenin, preventing its free presence in the cytosol until transcription is activated in the nucleus [31]. β-catenin activity is regulated by its phosphorylation on serine/threonine residues in the amino-terminal region of the protein [32].

In the absence of the Wnt ligand, free β-catenin in the cytosol is recruited by the adenomatous polyposis coli protein (APC) in a large cytoplasmic multiprotein complex named the “Destruction Complex”, formed by the proteins Axin, APC, casein kinase (CK1α), and glycogen synthase kinase 3β (GSK-3β). First, CK1α phosphorylates β-catenin on the Ser^45^ residue; then, GSK-3β phosphorylates β-catenin again on Ser^33^, Ser^37^, and Thr^41^, to be recognized by the E3 ubiquitin ligase β-TrCP complex and degraded by the 26S proteasome [33,34,35]. As a result, β-catenin cannot be translocated into the nucleus to activate the transcription of its target genes; instead, the members of the T cell factor/lymphocyte enhancer (TCF/LEF) transcription factor family associate with transcription inhibitors, forming complexes that prevent and block gene transcription [34,35].

Classically, the β-catenin pathway is activated by one of the so-called canonical Wnt ligands (Wnt-1, -2, -3, -3a, -8, and 8a). Upon binding the Frizzled (Fz) receptor and its coreceptors, the low-density lipoprotein receptor-related proteins 5/6 (LRP5/6), Wnt promotes the phosphorylation of LRP5/6 by CK1γ and GSK-3β, facilitating the activity of the cytoplasmic components Dishevelled (Dvl) and Axin. When Wnt binds Fz, Dvl is recruited by the latter through its PDZ domain and oligomerized in the plasmatic membrane, allowing the relocation of Axin and GSK-3β, thus promoting the phosphorylation of LRP5/6 and starting the signaling cascade [32]. Then, Dvl is phosphorylated by CK1γ and forms a complex with the GSK-3β binding protein (GBP) and Frat1, which inhibits the activity of GSK-3β. On the other hand, the formation of the Wnt/Fz-LRP-5/6 complex also promotes Axin degradation through LRP5/6.

Thus, a simultaneous inhibition of GSK-3β activity and Axin degradation prevents the Destruction Complex to be formed. As a result, β-catenin is not phosphorylated nor degraded. Free β-catenin can enter the nucleus and bind transcription factors of the TCF/LEF family; this results in a release of the Groucho protein, a transcriptional inhibitor of the TCF/LEF complex, and the recruitment of transcriptional coactivators like pygopus, B-cell lymphoma 9 (Bcl-9), the cyclic adenosine monophosphate (AMP) response element-binding protein (CBP), or its closely related homolog p300 [36,37], as well as other components of the basal transcription machinery. In turn, this activates the transcription of genes like *cyclin D1*, those coding for the Myc family of transcription factors—related to cell growth and proliferation and *axin2* [24,38,39] (Figure 1).

### 2.4. Crosstalk of β-Catenin Canonical Pathway

#### 2.4.1. Transcriptional Factors YAP and TAZ

It has been demonstrated that two transcriptional factors, the yes-associated protein (YAP) and tafazzin (TAZ), regulate the Wnt/β-catenin pathway. In turn, these factors are regulated by the Hippo signaling pathway, which controls tissue development, cell proliferation, and apoptosis [40]. When the Hippo pathway is activated, the mammalian aseptic 20 kinase (MST1/2) and Salvadoran homoloid 1 (SAV1) phosphorylate and activate LATS1/2. Activated LATS1/2 and MOB1 phosphorylate YAP or TAZ on serine residues, resulting in YAP/TAZ cytoplasmatic retention via its binding to the 14-3-3 protein, and its subsequent degradation in the cytoplasm. When the Hippo pathway is inactive, YAP/TAZ are activated and translocated to the nucleus, forming complexes with members of the TEA domain containing transcription factor family (TEAD) to mediate the transcription of genes that control stem cell maintenance, differentiation, cell proliferation, and apoptosis [40,41]. It has also been demonstrated that the tumor suppressor liver kinase B1 (LKB1) inhibits YAP by phosphorylation through the 5′-AMP-activated protein kinase (AMPK) [42].

Several works have reported the negative effect of YAP/TAZ on Wnt/β-catenin signaling. The phosphorylation of TAZ by the Hippo pathway sequesters Dvl, preventing the dissociation of the β-catenin Destruction Complex and inhibiting the β-catenin transcriptional activity [43]. Additionally, it has been reported that phosphorylated YAP binds β-catenin in the cytosol, preventing the activation of the Wnt signaling [44], and that YAP/TAZ is essential for the recruitment of the E3 ubiquitin ligase β-TrCP to the destruction complex and β-catenin degradation [45,46]. However, YAP is known to induce the activation of Wnt/β-catenin signaling by inhibiting GSK-3β, thus preventing β-catenin degradation [45]. Interestingly, it has been demonstrated that the Wnt/β-catenin pathway also regulates the activity of YAP/TAZ. When YAP is phosphorylated by the Hippo signaling, it is exported by Dvl to the cytoplasmic space, and this results in the inhibition of its transcriptional activity [43]. CD44, a cell surface glycoprotein, is transcriptionally regulated by β-catenin/TCF4 complexes [47]. The binding of hyaluronic acid to the CD44 receptor leads to an activation of tumor suppressor neurofibromin 2, which promotes the phosphorylation of Last1/2 by Mst/2, inhibiting YAP/TAZ [48].

An accumulation of β-catenin has been reported to promote p53 stability, inducing the activity of Lats 1/2 [24]. Furthermore, Azzolin et al. demonstrated that the β-catenin Destruction Complex is responsible for TAZ degradation by β-TrCP in the cytoplasm, where GSK-3β induces the phosphorylation of β-catenin, required for the interaction of TAZ with β-TrCP [46,49].

On the other hand, YAP is also a target gene for Wnt/β-catenin [45]. Wnt 3a induces TAZ dephosphorylation and stabilization, favoring the nuclear translocation of TAZ [49]. Interestingly, nuclear YAP can form a complex with β-catenin, resulting in a synergic effect between YAP/TAZ and β-catenin to induce either cell proliferation or apoptosis [50].

#### 2.4.2. Notch Signaling and Receptor Tyrosine Kinase Pathways

**Figure 2:**
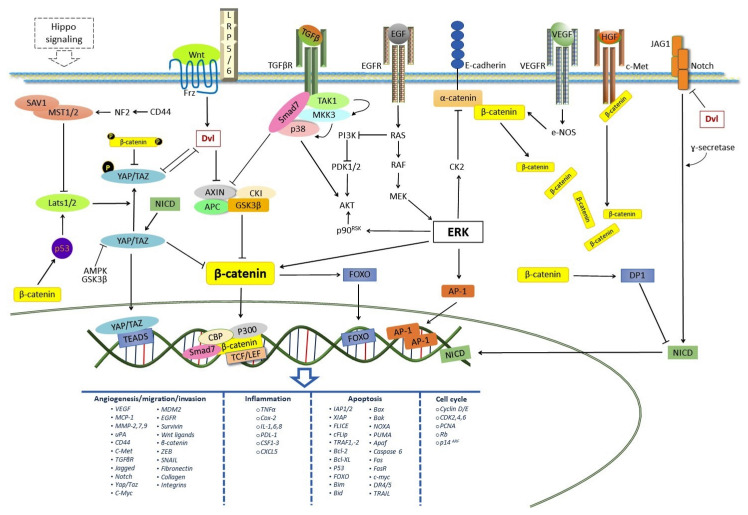
The crosstalk of the Wnt/β-catenin pathway with the Hippo, TGFβ, EGFR, VEGF, c-Met, and Notch signaling pathways modulates cancer progression and apoptosis, depending on the cellular context. An activation of the canonical Wnt pathway induces the stability of cytoplasmic β-catenin when the Wnt ligand binds FZ/Lrp5/6. This leads to a phosphorylation of Dvl, which inactivates the Destruction Complex, allowing an increase in the levels of free cytoplasmic β-catenin, which is then translocated to the nucleus, bound to TCF/LEF and transcription activators, to induce genic expression. On the other hand, the Hippo signaling increases β-catenin cytoplasmic levels by inactivating YAP/TAZ via SAV/MST1/2/LATS 1/2. When phosphorylated, YAP/TAZ activates the destruction complex via Dvl inhibition and sequestration of β-catenin. However, β-catenin also inhibits YAP/TAZ via Dvl and proteasomal activity, blocking the genic expression induced by YAP/TAZ. The Hippo signaling is activated via CD44/NF2 and p53. The TGFβ signaling pathway can also induce the cytoplasmic stability of β-catenin through smad 7. In turn, smad 7 interacts with TAK1 and MKK3, leading to the activation of p38 and the subsequent activation of AKT, inactivation of GSK 3β, and stabilization of β-catenin. The EGF pathway also can induce an increase in free cytoplasmic β-catenin by promoting the phosphorylation (inactivation) of α-catenin bound to E-cadherin through ERK activation. The VEGFR pathway also can increase the cytoplasmic levels of β-catenin through eNOS activation, which induces the S-nitrosylation of β-catenin, promoting its dissociation from α-catenin and its nuclear translocation. In addition, after the HGF ligand binds its receptor, c-MET promotes the phosphorylation of c-Met-associated β-catenin and a subsequent release of free cytoplasmic β-catenin. However, the activation of the Notch pathway by its Jagger ligand can decrease the cytoplasmic levels of β-catenin through the induction of DKK and Yap/Taz transcripts, which inhibit the Wnt/β-catenin pathway. Conversely, the Wnt/β-catenin pathway inhibits the Notch pathway through Dvl and DP1. Additionally, β-catenin induces the activation of the pro-apoptotic transcriptional factor FOXO. Depending on the cellular context, this could lead either to a carcinogenic process or a cell death process on cancer cells. Continue arrows (↓) indicate activation, arrows with (⊥) indicate inhibition.

While the above reports demonstrated the interactions between the Wnt/β-catenin and Hippo pathways and how their interplay contributes to homeostasis, tissue development, organ repair, tumorigenesis, and apoptosis, there is also a crosstalk between the Wnt/β-catenin and the Notch signaling and receptor tyrosine kinase pathways, such as EGFR, VEGF, TGF-β, and the hepatocyte growth factor (HGF) receptor-c-Met [51,52,53], which favor the stability of cytoplasmatic β-catenin. The binding of β-catenin to c-Met has been described. Upon binding HGF, c-Met induces the phosphorylation of β-catenin, with the ensuing the nuclear localization of the latter [51]. An activation of the EGFR pathway activates the RAS/RAF/MEK/ERK pathway. ERK leads to the dissociation of β-catenin from α-catenin/E-cadherin through CK2, resulting in the stability and nuclear translocation of β-catenin [52] (Figure 2)**.**

The TGF-β signaling pathway can also regulate the activity of β-catenin through the Smad proteins. Smad7 can directly bind Axin, inducing the inactivation of the Destruction Complex and stabilizing β-catenin; however, it also can inhibit GSK-3β [53]. VEGF-induced S-nitrosylation of β-catenin due to eNOS activation dissociates β-catenin from α-catenin or E-cadherin within adherens junctions, inducing its nuclear translocation and the activation of its target genes [54] (Figure 2). On the other hand, the binding of Notch to its Jagged (Jag) ligand causes the cleavage of the former, with the release and nuclear translocation of NICD, activating the expression of inhibitors of Wnt ligands like DKKs. It has also been observed that Notch binds (inactivates) β-catenin, activating the Destruction Complex and inducing the proteasomal degradation of β-catenin [55]. However, the Wnt/β-catenin pathway also inhibits the Notch pathway through Dvl and DP1 [56]. It has been reported that the expression of the genes coding for EGFR, c-Met, TGF-β, and Jag ligand is increased by the modulation of the Wnt/β-catenin signaling pathway [57,58,59,60] (Figure 2). On the whole, depending on the cellular context, the Wnt/β-catenin signaling and its crosstalk with other signaling pathways is thought to play a key role both in the regulation of cancer progression and in apoptotic death cell (Figure 2).

## 3. Apoptosis

Apoptosis, or programmed cell death, is a key component of biological processes like tissue homeostasis, embryonic development, differentiation, and defense against pathogens. Its dysregulation is known to cause many diseases, including cancer [61,62]. Various therapeutic strategies linked to the activation of apoptosis-related signal transduction pathways are used in clinical oncology, including irradiation, chemotherapy, immunotherapy, and suicide gene therapy [63,64,65]. Several of these strategies activate caspases, members of a large cysteine protease family. Caspases are synthesized as inactive forms called pro-caspases, and under external and internal stimuli they activate each other in a reaction cascade [66]. Caspases act as common death effector proteins in various forms of cell death, being capable of cleaving aspartate residue positions in numerous proteins involved in cell proliferation and survival, as well as deoxyribonucleic acid (DNA) repair. Apoptotic caspases are classified into two groups: Initiator caspases, which include caspase-2, -8, -10, and -9, and executioner (effector) caspases, including caspase-3, -6, and -7. When activated, initiator caspases cleave and activate executioner caspases, which lead to cell death. Two apoptotic pathways, named intrinsic and extrinsic, have been described. The caspase cascade is activated in both pathways, albeit at different entry points [67].

### 3.1. Extrinsic Apoptotic Pathway

The extrinsic pathway, also known as death receptor-mediated apoptosis, is activated by proteins of the tumor necrosis factor receptor (TNFR) superfamily, including CD95 (Fas), which binds the CD95 ligand (FasL) and by the tumor necrosis factor-related apoptosis-inducing receptor (TRAIR) [68,69]. Both death receptor types can activate apoptosis through a specific cytoplasmatic domain called death domain (DD). Death receptors are activated by binding their respective ligands, which results in receptor trimerization and the formation of a death-inducing signaling complex (DISC), which engages and then joins the adaptor molecule named Fas-associated death domain-containing (FADD) protein. The DISC-FADD complex binds initiator caspases like caspase-8 and -10 through their death effector domain (DED), causing the autocatalytic cleavage of procaspase-8. The activated caspase-8, in turn, activates executioner caspases (caspase-3 and -7). These proteases cleave cell death substrates, resulting in the morphological (cell junction disintegration, cytoplasm condensation, cell shrinking, and the formation of apoptotic bodies) and biochemical changes observed in apoptotic cells [70,71,72].

A crosstalk exists between the intrinsic and extrinsic apoptotic pathways, through caspase-8 and caspase-10; they cleave the proapoptotic Bid protein, which under normal conditions is located in the cytosol, but upon cleavage by caspase-8 or -10 produces a truncated Bid (_t_Bid) protein, which is then translocated to mitochondria, inducing mitochondrial outer membrane permeabilization (MOMP) with the subsequent release of cytochrome c (cyt c) from mitochondria into the cytosol. _t_Bid also induces the dimerization and translocation of the proapoptotic protein Bax from the cytosol to mitochondria, forming the Bax pore, through which cyt c is released, triggering mitochondrial apoptosis [73,74]. Cyt c binds and activates the apoptotic protease-activating factor 1 (Apaf-1), favoring the activation of the initiating caspase-9 and forming an apoptosome, which in turn activates caspase-3 [71,72,75].

**Figure 3:**
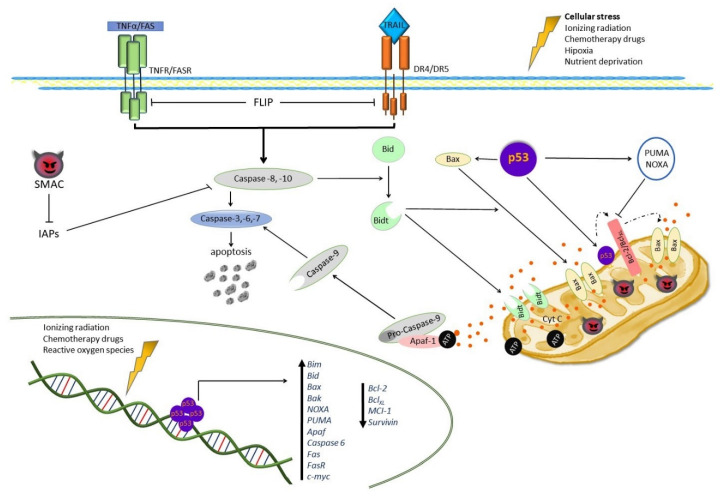
Extrinsic and intrinsic apoptosis pathway and its regulation via p53. Under cellular stress, the extrinsic pathway is started by ligand binding to death receptors, including TNFα, Fas and TRAIL; this leads to the autoactivation of caspases-8 and -10, which in turn promote the catalytic activation of the effector caspase-3. Another target of caspase-8 is the pro-apoptotic protein Bid, which is hydrolyzed to tBid, inducing Bax oligomerization and mitochondria depolarization with release of cyt c. Along with the activation of caspase-9, these events amplify the apoptotic pathway. The intrinsic pathway involves the permeabilization of the mitochondrial external membrane, which facilitates the cytosolic release of pro-apoptotic proteins like SMAC/Diablo and cyt c, which are otherwise confined within the intermembrane space. cyt c binds the Apaf-1 protein, which in turn binds and activates caspase-9, responsible for the activation of apoptosis executioners: Caspases-3, -6, and -7. On the other hand, SMAC/Diablo inhibits IAPs, which bind and neutralize caspases-8 and -10. Another protein involved in apoptosis regulation is p53, which transcriptionally activates pro-apoptotic genes and inhibits anti-apoptotic genes, directly inhibiting Bcl_XL_ and Bcl-2 in the mitochondria, favoring apoptosis. Continue arrows (↓) indicate activation, arrows with (⊥) indicate inhibition.

On the other hand, caspase-3 activation is inhibited by apoptosis inhibitors (IAPs) like c-IAP1, c-IAP2, Survivin, and X-linked IAP; in turn, these inhibitors are inhibited by the Second Mitochondria-derived Activator of Caspases (Smac)/Diablo. FLIP can be recruited to the DISC complex instead of caspases due to its sequence homology in the DED domains with caspase-8 and -10, and its expression levels have been correlated with chemotherapy resistance in cancer cells [71] (Figure 3).

### 3.2. Intrinsic Apoptotic Pathway

This programmed death cell pathway is activated by various cellular stress signals, like oxidative stress, DNA damage, ischemia, and increased intracellular Ca^2^ concentrations. These stimuli promote mitochondrial outer MOMP, which is regulated by the B-cell lymphoma 2 (Bcl-2) protein family. MOMP is blocked by the antiapoptotic members of the Bcl-2 family, Bcl-2, B-cell lymphoma-extra-large (Bcl_XL_), Bcl-W, and the myeloid cell leukemia protein 1 (Mcl-1), which inhibit the action of proapoptotic proteins of the same family, Bax-like proapoptotic proteins like Bax itself and Bak, as well as the proapoptotic BH3-only members, Bid, Bad, Bcl-2-like protein 11 (Bim), and p53-upregulated modulator of apoptosis (PUMA). This prevents the release of proapoptotic proteins like Smac, the high-temperature requirement A2 (Omi/HtrA2), and cyt c from mitochondria into the cytosol. In the cytosol, cyt c binds and activates Apaf-1, favoring the activation of caspase-9 and forming an apoptosome, which in turn activates caspase-3, -7, and -6, leading to apoptosis [71,76]. Smac/Diablo and Omi/HtrA2 antagonize cytosolic apoptosis inhibitors (members of the IAP family, like c-IAP1, c-IAP2, Survivin, and X-linked IAP), and thus promote apoptosis [77] (Figure 3).

### 3.3. Apoptosis Regulation via p53

The tumor-suppressing gene p53, also called the “the guardian of the genome”, is required to maintain genomic stability in mammalian cells [78]. Under normal conditions, p53 is inactivated by Mdm2 (E3 ligases) through protein-protein binding, promoting the ubiquitination and subsequent degradation of p53 [79]. Under stress conditions, Mdm is inactivated by p14^ARF^, breaking the bond between Mdm2 and p53 and promoting DNA repair, metabolic change, cell cycle arrest, and apoptosis. The nuclear transcription factor p53 can induce both the intrinsic and extrinsic apoptosis pathways, either through a mechanism that depends or not on its transcription activity. In the extrinsic pathway, p53 transactivates a death receptor like DR5 and the Fas ligand in response to DNA damage [80,81]. These receptors bind the p53-induced death-domain-containing protein (PIDD), which activates caspase-8 [62]. In the intrinsic pathway, p53 activates the transcription of proapoptotic proteins, including Bim, Bid, Bax, Bak, Noxa (also known as phorbol-12-myristate-13-acetate-induced protein 1, PMAIP1), PUMA, Apaf, and caspase-6 [82,83,84,85,86,87] while it trans-represses antiapoptotic genes like *Bcl-2*, *Mcl-1* [88,89], and *Survivin* [90]. On the other hand, p53 has been reported to induce apoptosis independently of its transcriptional activity [91]. A cytosolic accumulation of p53 induces the direct activation of Bax, favoring MOMP, with the subsequent release of cyt c and caspase activation [92]. The mitochondrial translocation of p53 induces apoptosis by interacting with Bclx_L_ and Bcl-2, inhibiting the sequestration (inactivation) of antiapoptotic proteins by proapoptotic Bcl-2 members [93]. Additionally, mitochondrial p53 directly promotes the MOMP-inducing activity of Bax or Bak [94,95]. Furthermore, it has been reported that p53 induces apoptosis by increasing the levels of the transcriptional factor c-myc [96]. C-myc promotes apoptotic cell death in response to several stimuli, including DNA damage, hypoxia, and a depletion of survival factors [97,98,99] by lowering the levels of Bclx_L_, Bcl-2, and FLIP [100,101] and increasing the expression and activity of Bid, Bim, Bax, Bak, Noxa, and Puma [102,103,104] leading to the activation of caspases -8, -9, and -3 [99,105] (Figure 3).

Furthermore, it has been demonstrated that c-myc can induce apoptosis by activating the transcription and signaling of Fas/FasR, TNFα, and the TNF-related apoptosis-inducing ligand (TRAIL) [99,106,107,108]. In addition, c-myc induces apoptosis through p53 [99].

## 4. Proapoptotic Effect of the Wnt/β-Catenin Pathway in Cancer Cells

The link between the Wnt/β-catenin pathway and cell processes like apoptosis and proliferation can be demonstrated by the fact that inhibiting and overexpressing β-catenin promotes apoptosis and inhibits proliferation, respectively, in several mouse models and in mammalian cell lines. In the following sections, we will discuss the findings of various studies that demonstrated the proapoptotic effect of β-catenin in several cancer models.

### 4.1. Glioblastoma

Malignant tumors in the central nervous system (CNS) usually have a poor prognosis, with high morbidity and mortality rates, severely affecting the patient’s cognitive function and quality of life. Malignant gliomas are the most common type of primary brain tumor, with a yearly incidence of 5.26 cases per 100,000 population [109]. Among them, glioblastoma multiforme (GBM) is the most aggressive type; it is characterized by high mitotic activity rates, necrosis, inflammation, cell proliferation, and thrombosis. Glioblastoma multiforme can occur in the novo as a grade 4 neoplasia or have a malignant progression from a low- to a high-grade. These tumors show an infiltrative growth pattern that confers them a high resistance to treatment, yielding mean survival times of 12–15 months after diagnosis [110].

Recent studies suggest that the Wnt/β-catenin signaling pathway could regulate tumor growth in glioma [111] and that manipulating components of the Wnt pathway could suppress the growth of malignant gliomas [112,113,114]. Treatment with sulforaphane potentiates the proapoptotic effect of temozolomide (TMZ) on glioma cells by enhancing the activity of caspase-3 and -7 and the expression of Bax, by lowering the levels of miR-21 through Wnt/β-catenin signaling [115].

Other studies showed the proapoptotic role of β-catenin in the reduction of resistance to TMZ by cordycepin on glioma cells, by inhibiting O^6^-methylguanine-DNA methyltransferase (MGMT), as well as by depleting glutathione (GSH) and generating reactive oxygen species (ROS) [116].

The expression levels of MGMT, a DNA-repairing protein, have been found to correlate inversely with chemosensitivity and a better prognosis in glioblastoma patients [117]. GSK-3β inhibition also sensitized glioblastoma multiforme cell lines to TMZ [118]. V. Pyko et al. suggested that GSK-3β inhibition enhances the antineoplastic effect of TMZ on T98G, U138, U251, and U87 human glioma cells by decreasing the expression of MGMT via its c-myc-mediated promoter methylation [119]. Those authors suggested that c-myc binds the MGMT promoter, which results in the recruitment of DNA (cytosine5)-methyltransferase 3A, a methylation of the MGMT promoter, and the silencing of MGMT expression.

The Wnt/β-catenin pathway has been reported to regulate the transcription of c-myc [119,120]. Thioridazine (an antipsychotic drug) induces autophagy in glioblastoma multiforme cells by upregulating the activity of AMPK, enhancing p62-mediated autophagy and apoptosis through Wnt/β-catenin signaling [121]. In addition, co-administering Thioridazine and TMZ enhanced autophagy, inducing apoptosis in glioma cells [121].

On the other hand, simultaneously treating U87 glioma cells with an AZD2858 (a GSK-3β inhibitor) and ICG-001, a histone acetyl transferase inhibitor that antagonizes Wnt/β-catenin/Tcf-mediated transcription by binding CBP, reduced cell proliferation and increased the cell fraction in the G_0_/G_1_ phase of the cell cycle; these effects were significantly higher in the group treated with ICQ-001 [111]. Furthermore, AZD2858 inhibited migration and invasion in U87 cells [111]. Those authors indicated that the antineoplastic effect observed by activators and inhibitors of the Wnt/β-catenin pathway on U87 glioma cells follow different mechanisms, due to a differential genic expression in both groups [111].

Activating the Wnt/β-catenin signaling pathway could reduce proliferation, migration, and invasion in glioma cells by downregulating cancer-associated pathways [111]. AZD2858 downregulated the TGF-β signaling by reducing the levels of the TGF-β receptor 1 (TGFβR1), as well as Smad 2 and Smad 3 [111]. AZD2858 downregulated the cell cycle pathway by decreasing the expression of cyclins like A2 and B3, and the cyclin-dependent kinase 1 (CDK1) [111]. Furthermore, this activator inhibited the expression of CD44, the collagen type I alpha 1 chain (Col1A1), the laminin subunit gamma 3 (LAMC3), TGFBR1, the vascular endothelial growth factor D (VEGF-D), the ETS proto-oncogene 1 (ETS1) and Survivin [111]. In addition, AZD2858 induced the upregulation of signaling pathways like Hippo and p53, which play a key role in cell cycle arresting and in inducing apoptotic cell death by increasing the levels of p21, the growth arrest, and DNA-damage-inducible protein (GADD45G) gamma, sestrin 3, the Fas cell surface death receptor, and caspase-3. On the other hand, ICG-001 upregulated the expression of the lysosome pathway, enhancing the levels of cathepsin C, glucosidase alpha, tripeptidy1 peptidase 1 (TPP1), N-acetyl-alpha-glucosaminidase, glucosylceramidase β, mannosidase β, the GM2 ganglioside activator, and prosaposin [111].

In conclusion, it has been suggested that activating the Wnt/β-catenin signaling pathway could reduce the survival and invasion of U87 cells by inhibiting GSK-3β [111]. Lithium chloride (LiCl, a GSK-3β inhibitor) was reported to reduce invasion rates in the U87 glioma line while increasing β-catenin activity [122,123].

Reis et al. reported that activating the endothelial Wnt/β-catenin signaling by Wnt-1 reduced angiogenesis, decreased tumor growth, and partially resolved the BBB disruption in glioma models by inducing the expression of delta-like 4 (Dll4) and the platelet-derived growth factor B (PDGF-B), resulting in the activation of the Notch signaling and increasing the coverage of smooth muscle cells and pericytes [124].

Tan et al. reported that the dapper homolog 2 (DACT2), a tumor suppressor, is under-expressed in glioma tissues, and its expression was negatively correlated with glioma grade and a poorer survival, while its overexpression in U87 and U251 glioma cells induced arrest of the cell cycle in the G_0_/G_1_ phase, reducing cell proliferation and promoting apoptosis through a downregulation of PCNA and cyclin D1, while increasing the levels of the proapoptotic protein Bax and the pYAP transcription factor [125]. When the YAP is phosphorylated, it is retained in the cytoplasm by its binding to the 14-3-3 protein, and subsequently it is degraded by the proteasome [126]. Those authors suggest that the YAP transcription factor is inhibited by DAC2 through the Wnt/β-catenin pathway [125,126]. Lee et al. reported that Dvl binds phosphorylated YAP, suppressing its nuclear translocation and consequently its transcriptional activity [127]. Furthermore, LKB1/AMPK and p53/Lats2 were proved to be necessary for the inhibition of nuclear YAP by Dvl [127]. An upregulation of YAP has been reported in glioma cells, linked to a lower overall patient survival [128].

These results demonstrate that the modulation of the Wnt/β-catenin pathway can induce apoptotic cell death and autophagy through different mechanisms, and that the modulation (either activation or inhibition) of the Wnt/β-catenin pathway in multiple glioblastoma can induce apoptotic cell death and autophagy through the regulation of AMPK, c-myc, Hippo, and the p53 signaling pathway.

### 4.2. Colorectal Cancer

Colorectal cancer is the third most deadly and fourth most commonly diagnosed cancer in the world, with an incidence of 11% and a mortality rate of 5.8% among all cancer types [129]. An estimate of 1.8 million new cases occurred in 2018, with a mortality of 0.88 million. A secondary metastatic disease worsens the prognosis, with a median 5-year survival of 18.5% in the USA and 27.7% in Europe [129]. Standard therapy for colorectal cancer includes surgery, chemotherapy, and radiotherapy [130]. These treatments can be combined, depending on the location and progression of the disease. About 54% of patients relapsed, even after neoadjuvant treatment [130].

A dysregulated Wnt/β-catenin signaling pathway, usually stemming from mutations in the genes coding for adenomatous polyposis coli and β-catenin, leads to colorectal tumors [131]. Dietary fiber has shown a protective effect against colorectal cancer [132]. Lazarova et al. found that butyrate, a histone deacetylase (HDAC) inhibitor derived from the fermentation of dietary fiber in the colonic lumen, induces apoptosis in colorectal cells, in correlation with the degree of Wnt/β-catenin signaling pathway hyperactivation. Those authors suggested that both higher and lower levels of Wnt/β-catenin activity can lead to apoptosis in colorectal cancer cells, while moderate levels of dysregulated Wnt/β-catenin activity promote proliferation [132,133]. They have identified two classes of colorectal cancer cell lines: Those that respond to butyrate treatment with a high-fold increase in Wnt activity and apoptosis (HWA), and those that exhibit a low-fold induction of Wnt activity and apoptosis (LWA). Thus, they suggest that the heterogeneity observed in the levels of Wnt signaling in different colorectal cancer cells in vitro may be analogous to the presence of a significant heterogeneity in colorectal tumors in vivo [132].

Bordonaro et al. showed that other HDAC inhibitors (HDACis), such as TSA, SAHA, and MS275, induced apoptosis in HCT-116 cells, and that the exogenous expression of Dkk-1, an antagonist of the Wnt/β-catenin pathway, suppressed the induction of Wnt transcriptional activity and apoptosis by HDACis through an increase in Ser^37^ and Thr^41^ phosphorylation of β-catenin [134]. An increased induction of the Wnt signaling pathway at the plasma membrane by HDACis could be due either to an enhanced expression of Wnt ligands and/or their receptors; a reduced expression of Wnt signaling inhibitors acting at the ligand level; alterations of Wnt ligands and/or their receptors; and/or a higher secretion of Wnt ligands or a higher presence of their receptors on the cell surface [134].

The apoptotic effect of butyrate on colorectal cancer cells can be further increased by propolis, a honeybee product [135]. The hyperactivation of Wnt signaling by butyrate is influenced by the CREB-binding protein (CBP) and p300, histone acetylases that associate with β-catenin [136]. A modulation of CBP- or p300-mediated Wnt signaling affects the ability of butyrate to induce Wnt activity and apoptosis [137,138]. An inhibition in the association between CBP and β-catenin by ICG-001, a histone acetyl transferase inhibitor that binds CBP but not p300, significantly repressed butyrate-led hyperactivation of Wnt/β-catenin signaling in SW620 (p300-deficient) and HCT-116 (p300-expressing) cells [139]. ICG-001 does not interfere with butyrate-induced apoptosis in HCT-116 cells; instead, a combination of butyrate/ICG-001 potentiated apoptosis [139]. However, ICG-001 did interfere with butyrate-induced apoptosis in SW620 cells, resulting in cell cycle arrest in the G_1_ phase and the up-regulation of p21 [139]. Furthermore, butyrate significantly reduced the levels of the antiapoptotic protein Survivin in HCT-116, but not in SW620 cells [139]. The differences in Survivin expression may also contribute to the differential effects of the butyrate/ICG-001 combination on apoptosis in HCT-116 and SW620 cells [139].

On the other hand, p300 was proved to promote the induction of Wnt/β-catenin signaling activity by butyrate, and that treatment with ICG-001 interfered with the ability of p300 of enhancing butyrate-mediated Wnt hyperactivation [137]. CBP and p300 have shown differential roles in CBP- and p300-mediated Wnt signaling in colon cells. CBP-Wnt activity promotes cell proliferation, while p300-Wnt activity is associated with cell differentiation and, possibly, apoptosis. Treating cells with ICG-001 enhances the association between p300 and β-catenin at the expense of the CBP/β-catenin association, promoting cellular pathways that favor differentiation and/or apoptosis while repressing cell proliferation [136,140]. Thus, ICG-001 inhibits cell proliferation in colorectal cancer, increases apoptosis (as measured by caspase activity), and inhibits Survivin expression [136,140]. A water-soluble version of ICG-001 reduced the formation of intestinal neoplasms in the APCMin mouse model of colorectal cancer [136]. In addition, butyrate-treated HCT-R cells failed to show Wnt signaling hyperactivation, exhibiting lower levels of apoptosis and increased cell growth compared to parental HCT-116 cells; however, they were extremely sensitive to ICG-001-induced apoptosis, suggesting that the survival of butyrate-resistant cells is associated with a CBP-mediated Wnt activity [137]. Meanwhile, the sensitivity to butyrate in HCT-R, D10, and F5 cells was restored by reintroducing p300 expression [141], increasing p300-mediated Wnt/β-catenin activity [137]. An overexpression of nuclear p300 is associated with a more favorable prognosis (disease-free survival rate) specifically in colon, but not rectal, cancer [142].

The ZEB1 transcriptional factor has been reported to mediate drug resistance and the epithelial–mesenchymal transition in p300-deficient colorectal cancer cells [143]. When stimulated by APC inactivation, Wnt signaling synergizes retinoblastoma (Rb) inactivation to induce apoptosis through a mechanism mediated by increased activity of the target of rapamycin complex 1 (TORC1), causing metabolic, oxidative, and energy stress [144]. On the one hand, by enhancing Wnt activity, butyrate may induce apoptosis during Rb inactivation (in the G_1_-S transition); meanwhile, by blocking the cell cycle and promoting Rb hypophosphorylation, butyrate represses the synergy between Wnt activation and Rb inactivation [145].

p300 has also been reported to interact with Rb, modulating the cell cycle progression in colorectal cancer cells [146]. Unphosphorylated (active) Rb typically halts the cell cycle, while inactivating Rb phosphorylation allows the cycle to resume and cell proliferation to progress [147]. Additionally, butyrate has exhibited strong anti-inflammatory properties, and this effect is likely mediated by inhibiting the production of TNF-α, the activation of the nuclear factor kappa-light-chain-enhancer of activated B cells (NF-κB), and the expression of IL-8, -10, and -12 in immune and colonic epithelial cells [142,148].

On the other hand, a three-fold increase in apoptosis rates was observed when HCT-116 cells (*KRAS* mutant) were incubated at 42 °C, from 10.8% ± 0.6% (37 °C) to 31.8% ± 2.6% (42 °C); this was due to an increase in the levels of the epidermal growth factor receptor (EGFR), which led to a sustained hyperactivation of the extracellular-signal-regulated kinase (ERK) and higher Wnt/β-catenin transcription activity, that resulted in higher levels of E-cadherin, FRA1, and c-JUN, along with a downregulation of c-myc and Matrix metallopeptidases (MMP) 2 [149].

These studies also indicate that suppressing the activity of ERK1 decreases the levels of Wnt/β-catenin during hyperthermia, and the induction of Wnt/β-catenin activity with GSK-3β inhibitors leads to increased levels of the pERK ½ [149]. A positive crosstalk between the activity of tyrosine kinases/KRAS/ERK and WNT/β-catenin would result in the hyperinduction of the transcriptional activity of Wnt/β-catenin above optimal levels for cell proliferation, favoring the apoptotic process in colorectal cancer cells [149]. ERK activation promotes Wnt/β-catenin signaling by increasing β-catenin nuclear levels, partly by downregulating E-cadherin [150]; EGFR has been proposed to act as a transcriptional target of Wnt/β-catenin signaling [151]. EGF induces the activation of ERK, which phosphorylates (inactivates) α-catenin through the casein kinase (CK)-2, promoting its dissociation from β-catenin and the transactivation of β-catenin [52]. Furthermore, it has been reported that ERK induces the activation of p90^RSK^ and the phosphorylation of GSK-3β on Thr^43^, subsequently facilitating the phosphorylation on Ser^9^ of GSK-3β by p^90RSK^, resulting in the inactivation of GSK-3β and the activation of β-catenin [152].

Missense *KRAS* mutations have been found in 40%–45% of colorectal cancer patients, while mutations affecting the activity of Wnt/β-catenin were detected in over 80% of colorectal cancer patients [153]. Furthermore, it has been reported that a downregulation of β-catenin caused a drop in rhTRAIL sensitivity in the Ls174T human colon carcinoma cell line, by downregulating the expression and translocation to the cell membrane of the death receptors DR4 and DR5, independently of TCF-4-signaling [154].

It has been suggested that β-catenin can also interact with MUC1, the receptors of growth factors like c-Met and c-erbB2, and the forkhead box class O (FOXO) transcription factors, which could directly or indirectly influence the expression of DR4 and DR5 [155,156,157]. The phosphorylation (inactivation) of Foxo3a was inhibited by activators of the Wnt/β-Catenin pathway, like LiCl and Wnt3a [158]. Furthermore, the armadillo repeats of β-catenin have been reported to interact with FOXO, promoting its transcriptional activity [159]. FOXO regulates the transcription of the proapoptotic genes *FASL*, *TRAIL*, *DR4*, *DR5*, *Bim*, and *Puma*, and it also induces apoptosis by a p53-dependent mechanism [160].

Nonactin (a ionophore macrotetrolide antibiotic) [161] induced caspase-dependent apoptosis in β-catenin-mutated (active) colon tumor cell lines, including HCT 116, LS174T, and SW48 cells, by inhibiting the glycolytic pathway and promoting the expression of mitochondrial uncouplers that induced a loss of mitochondrial membrane potential. A significant tumor regression has also been observed in a xenograft model based on the β-catenin-mutant HCT 116 cell line [162], but not in the β-catenin wild-type A375 xenograft model, in response to nonactin.

Mutant β-catenin was suggested to bind and modulate the activity of proteins that participate in glycolysis, thereby disrupting the Warburg effect [162]. β-catenin phosphorylation on Tyr^333^ by c-Src facilitated its binding to nuclear pyruvate kinase M2 (PKM2), promoting a regulation of the Warburg effect [163]. Christofk et al. reported that binding of PKM2 in the nucleus to phosphotyrosine peptides results in a release of the allosteric activator fructose-1,6-bisphosphate, leading to an inhibition of the enzymatic activity of PKM2 and consequently a decreased glycolytic activity [159,164].

These results suggest that the Wnt/β-catenin pathway induces apoptosis in colorectal cancer cells by modulating the EGFR/RAS/RAF/MEK/ERK pathway, and during Rb inactivation. Additionally, the Wnt/β-catenin pathway can induce cell death by regulating cell metabolism and the expression of receptor deaths, possibly through FOXO [164].

### 4.3. Osteosarcoma

Osteosarcoma is a malignant, primary bone tumor, common in children and young adults. It accounts for about 20% of all benign and malignant bone neoplasms, and 2% of all pediatric cancers [165]. A second incidence peak is observed in adults older than 65 years. Osteosarcoma can develop in any bone, but it chiefly affects long-bone metaphysis (distal femur > proximal tibia > proximal humerus); the axial skeleton and skull are often involved in elderly patients. The clinical diagnosis of osteosarcoma relies on the finding of malignant osteoblasts and their products, immature bone or osteoid tissue. The long-term survival rate after chemotherapy and surgery is 70%. Patients with metastatic, mainly lung disease, show 5-year survival rates of about 40% or less [166].

Previous studies showed that the canonical Wnt/β-catenin pathway was inactivated in bone and soft-tissue sarcoma cells, including osteosarcoma [167]. This observation suggested a tumor-suppressing function for the Wnt/β-catenin pathway in osteosarcoma [168].

The concentration of zinc in the tissues and blood of osteosarcoma patients has been found significantly reduced with respect to healthy subjects [169]; further studies reported that zinc administration showed an antineoplastic effect, causing a drop in proliferation and invasion rates and leading to caspase-3 and- 9-related apoptotic cell death in U-2OS osteosarcoma cells, activating the Wnt/β-catenin pathway by increasing the levels of Wnt3 and β-catenin [168].

MG132 (a proteosomal inhibitor) simultaneously promoted the expression of the proapoptotic protein Noxa and the nuclear translocation of β-catenin in the Saos2 osteosarcoma cell line through a p53-independent pathway, favoring caspase-3 activation [170]. These results also suggested a link between β-catenin-mediated signaling and apoptosis induction; it was already known that β-catenin is responsible for inducing *Noxa* mRNA [170]. Noxa interacts with antiapoptotic members of the Bcl-2 family, and it causes the release of cyt c into the cytosol, leading to caspase activation and the ensuing caspase-dependent apoptosis [171]. In addition, it was demonstrated that MG132 induces apoptosis on Saos-2 cells through ROS generation, a loss of mitochondrial transmembrane potential and caspase-3 activation, with a modest decrease in the levels of the antiapoptotic proteins Bcl-2 and Bclx_L_ [172].

Suppressing the long non-coding RNA HNF1A antisense RNA 1 (HNF1A-AS1) has shown antitumor effects, inhibiting cell proliferation, invasion, and metastasis, while inducing apoptosis on the MG-63 osteosarcoma cell line [173]. It was suggested that HNF1AAS1 might act as an oncogenic lncRNA that boosts proliferation and metastasis in osteosarcoma cell lines and activates the Wnt/β-catenin signaling pathway [173]. The expression levels of HNF1AAS1 were significantly higher in osteosarcoma-derived tissue compared with adjacent non-tumor tissues, and it is associated with a poor prognosis in osteosarcoma patients [173]. Liu et al. showed that lncRNA HNF1A-AS1 acted as an oncogene and autophagy promoter in hepatocellular carcinoma through hsa-miR-30b-5p sponging [174].

It has also been demonstrated that activating Wnt/β-catenin signaling reverses the resistance to gemcitabine (a DNA synthesis inhibitor) and induces apoptosis by decreasing Beclin-1 expression and inhibiting autophagic cell death in the MG63 osteosarcoma cell line [175]. Previous studies reported that Beclin-1 is required for the initiation and progression of autophagy, and that autophagy activation may inhibit the ability of antineoplastic drugs to induce apoptosis [176,177]; it was known that autophagy can be a survival mechanism in cancer cells [110,178], and the Wnt/β-catenin signaling pathway is a negative autophagy regulator [179,180,181].

Petherick et al. reported that β-catenin is a transcriptional corepressor of TCF4, which inhibits the transcription of p62/SQSTM1 (Sequestosome 1) and autophagosome formation under normal conditions and during nutrient deprivation [182]. Furthermore, it has been demonstrated that inhibiting the Wnt/β-catenin pathway induces the overexpression of p62, leading to autophagy through the activation (dephosphorylation) and nuclear translocation of the transcription factor TFEB, inactivating the mammalian target of rapamycin (mTOR) [183]. Shimozaki et al. demonstrated that inhibiting the activity and expression of GSK-3β suppressed proliferation and induced apoptosis in the HOS, MG-63, and Saos-2 osteosarcoma cells lines; it also reduced the growth of orthotopic osteosarcoma in mice [184]. The therapeutic efficacy of GSK-3β inhibition was suggested to be associated with an increased expression, nuclear location, and co-transcriptional activity of β-catenin.

These results suggest that the Wnt/β-catenin pathway induces apoptosis in osteosarcoma cells by modulating the proteasomal and autophagy pathways, among other mechanisms [184].

### 4.4. Melanoma

Melanoma is the skin neoplasm with the highest impact on public health worldwide. In 2016, 82,476 new melanoma cases were reported in the USA, while about 7200 deaths due to melanoma were reported in 2019 [185]. Patients diagnosed with primary melanoma have a 5-fold higher risk of developing invasive melanoma with respect to the general population [186]. The early signs of melanoma include asymmetric lesions with irregular edges, a mottled color, ≥6 mm in diameter, and/or the rapid development of a new lesion, particularly in adults. A cutaneous lesion that looks different from surrounding marks is an important finding in patients with multiple nevi. A definitive diagnosis for melanoma relies on histopathological studies [187].

A correlation between decreased survival rates and lower nuclear β-catenin levels was observed in biopsy samples from melanoma patients, suggesting that the loss of Wnt/β catenin signaling plays an important role in melanoma pathogenesis [188,189]. Transgenic mouse models expressing a melanocyte-specific, constitutively active mutant β-catenin failed to display spontaneous melanomas. Furthermore, high levels of cytoplasmic and/or nuclear β-catenin in melanoma tumor biopsies correlated with a better patient survival, decreased metastasis recurrence, and a decreased expression of the cell proliferation marker Ki-67 [190].

The levels of DKK1, an inhibitor of the WNT/β-catenin pathway, are notably reduced in melanoma cells compared with melanocytes [191]. An activation of Wnt/β-catenin signaling in B16-F1 melanoma cells due either to a lentivirus-led expression of WNT3A (B16:WNT3A) and/or pharmacological GSK-3β inhibition results in significantly reduced proliferation rates with respect to B16:GFP cells, along with an upregulation of *axin2* [190]. Additionally, the activation of the Wnt/β-catenin pathway by WNT3A led to an upregulation of Trpm1, Stem cell-factor (Kit), Met, and Mlana [190]. The loss of TRPM1 has been linked to decreased survival rates and an increased risk of metastasis [192].

On the other hand, the effect of an inhibition of BRAF-mediated signaling in melanoma was studied by co-administering WNT3A and PLX4720 (a BRAF^V600E^ inhibitor). Biechele et al. showed that an activation of the Wnt/β-catenin pathway and PLX4720 synergistically induced apoptotic cell death in BRAF^V600E^ mutant melanoma cell lines by increasing the activity of the proapoptotic isoforms Bim_L_ and Bim_S_, as well as caspase-3 [193]. Meanwhile, this cotreatment inhibited the autophosphorylation of GSK-3β on Tyr^216^, which is associated with a stabilization of β-catenin and decreased levels of Axin1, a negative regulator of the Wnt/β-catenin pathway [193]. A drop in Axin1 levels was also observed to precede and be independent of apoptosis onset, significantly inhibiting tumor growth in melanoma mouse models induced by WNT3A and PLX4720.

It was suggested that the activation of Wnt/β-catenin signaling and the decreased levels of Axin1 can activate apoptosis by inhibiting BRAF [194]. WNT3A was demonstrated to induce dephosphorylation [195] and subsequent degradation of Axin1. In the absence of a Wnt ligand, GSK-3β is activated and phosphorylates Axin1, improving its stability [193]. Treating WNT3A-expressing melanoma spheroids with PLX4720 led to a decrease in spheroid size and in the number of invasive cells with respect to PLX4720-treated, GFP-derived spheroids or DMSO-treated, WNT3A-derived spheroids [194].

Nearly 45% of melanoma lines showed *BRAF*-activating mutations, and 20% showed *NRAS*-activating mutations; both mutations activate the ERK/MAPK (mitogen-activated protein kinase) pathway [196]. A differential sensitivity was observed with simultaneous treatment of *NRAS*-mutant melanoma cells with WNT3A and AZD6244 (a MEK inhibitor); cell lines sensitive to apoptosis such as SK-MEL-2 and A375 showed an increased activation of the Wnt/β-catenin pathway and higher Axin2 levels, along with a decrease in Axin1 via proteosome through ERK inhibition [197]. Apoptosis can be induced in M202 and M207 apoptosis-resistant *NRAS*-mutant melanoma lines by treatment with AZD6244, via Axin1 depletion [197]. These results suggest that Axin1 plays a key role in regulating the apoptotic process in *NRAS-* and *BRAF*-mutant melanoma lines [197]. Targeted BRAFV600E inhibitors like PLX4032 (vemurafenib) [198], PLX4720 [199], and GSK2118436 have shown a high response rate in patients with BRAFV600E tumors [200] [201]. Phase 1 clinical data revealed a remarkably high response rate (81%) in metastatic melanoma patients orally treated with 960 mg twice a day of a BRAFV600E inhibitor [202]. These data demonstrate that *BRAF*-mutant melanoma cells are highly dependent on the activity of B-RAF kinase [202].

It has also been reported that an activation of the Wnt/β-catenin signaling in WNT3-conditioned medium increased apoptotic cell death rates promoted by the recombinant human TNF receptor death-inducing ligand (rhTRAIL) in melanoma cells, by increasing the levels of the Bim and PUMA proapoptotic proteins, reducing the levels of the Mcl1 antiapoptotic protein, and upregulating mRNA expression for genes like *axin2, LEF1,* and *TNFRSF19* [203]. Furthermore, boosting the β-catenin pathway by CHIR99021 (a GSK-3 inhibitor) or by *axin1* siRNA significantly sensitized melanoma cells to rhTRAIL [203]. Conversely, β-catenin siRNAs inhibited apoptosis [203]. It was suggested that targeting Axin1 stability by post-translational modifications like phosphorylation by GSK-3β, PARsylation by tankyrase-1/2 (TNKS1/2), methylation by protein arginine methyltransferase 1 (PRMT1), and SUMOylation [193,204,205] could sensitize melanoma cells to TRAIL-based therapies [203].

In lung cancer lines, β-catenin induced the relocation and redistribution of death receptors like DR4 and DR5 on the cell membrane, inducing TRAIL-mediated extrinsic and intrinsic apoptosis by activating caspase-8 and -3 [206]. A loss of membrane-bound β-catenin, reported in tumor cells with cytoplasmic and nuclear β-catenin location, may favor the expression of proapoptotic DRs [207].

Arozarena et al. demonstrated that the expression of β-catenin and the melanocyte-specific transcription factor (MITF) are significantly reduced in areas of dermal invasion within primary tumors, and that an accumulation of cytoplasmic/nuclear β-catenin, which correlated with MITF expression levels in melanoma cells, inhibited invasion into 3D collagen systems [208]. Inhibiting MITF with siRNA in melanoma cells induced a strong increase in the invasive activity in 3D collagen, suggesting that MITF suppresses the invasive capacity of β-catenin by blocking the rho-associated kinase (ROCK)-regulated cell morphology in invading WM266-4 melanoma cells, while interfering with β-catenin-induced expression of MT1-MMP, which activated proMMP-2 [208]. MITF was suggested to recruit β-catenin, and due to this recruitment and interaction, MITF could interfere with the regulation of β-catenin at TCF/LEF-target genes like MT1-MMP [208].

Docosahexaenoic acid (DHA, polyunsaturated fatty acid) was proved to reduce the proliferative and invasive potential, inhibiting the gelatinolytic activity of MMP-13, MT1-MMP, and MMP-2 in B16F10, WM115, and WM266-4 melanoma cells, by increasing the nuclear relocation of pSer^675^ β-catenin by a PKA-dependent pathway, while increasing MITF levels [209].

In fetal kidney HEK293 cells, β-catenin phosphorylation on Ser^675^ reduced its proteasomal degradation and increased its nuclear expression [210]. DHA also induced caspase-3-dependent apoptosis in melanoma lines by increasing the Bax/Bcl-2 ratio [211]. These results clearly show that the modulation of the Wnt/β-catenin pathway can induce extrinsic and intrinsic apoptosis in melanoma cells through its ability to interact with other signaling networks involved in the regulation of cell death mechanisms [211].

### 4.5. Medulloblastoma

Medulloblastoma is one of the most frequent malignant brain tumors in the pediatric population. Age on diagnosis is usually 6–8 years, although it may occur in adult patients (the incidence is 0.05 cases per 100,000 population). Histologically, it is a cerebellar embryonic tumor. It is more common in male than in female patients, with a male–female ratio of 1.8: 1 [212]. Transcriptomic, molecular, and genetic studies, along with clinical traits, suggest the existence of four distinct medulloblastoma subgroups in both pediatric and adult populations: WNT, sonic hedgehog (SHH), Group 3, Group 4 [213,214].

WNT medulloblastoma, which accounts for 10% of all medulloblastomas, shows a loss of chromosome 6 and activating mutations in the CTNNB1 gene, encoding for β-catenin [213]. Clinically, WNT medulloblastomas have the best prognosis, with a 5-year survival of 95% [215]. A better outcome for this group has been confirmed by other studies, citing long-term survival exceeding 90% in WNT patients [9,216,217,218,219,220,221]. On the other hand, SHH, Group 3, and Group 4 medulloblastoma subtypes are characterized by metastasis and increased rates of recurrence, resulting in an intermediate-poor overall prognosis and survival. SHH medulloblastoma patients show an intermediate long-term survival rate, whereas Group 3 and -4 patients have similar outcomes [215]. The SHH group, found in 30% of all medulloblastoma cases, is vital for the proliferation of cerebellar granule neuron precursors (GNPs) [222]; its constitutive activation triggers medulloblastoma [223].

While the canonical Wnt/β-catenin pathway is required for GNPs maturation, a constitutive activation of this pathway inhibits cell proliferation of GNPs [224]. Poschl et al. reported that activating the Wnt/β-catenin pathway inhibits SHH signaling in ctnnb(ex3)Fi/+ cerebellar GNP, decreasing cell proliferation and the oncogenic capacity in GNPs induced by SmoM2 (ctnnb(ex3)Fi/+ SmoM2 Fi/+) [225].

In addition, the activation of the Wnt/β-catenin pathway in Math1:SmoM2 Fi/+ ctnnb(ex3)Fi/+ mice (a well-known Shh-medulloblastoma model) significantly decreased medulloblastoma formation rates, resulting in a decreased cerebellar size [225]. LiCl (a GSK-3β inhibitor) notably decreased cell proliferation in cerebellar GNPs in Math1::SmoM2 Fi/+ neoplastic cells and in neoplastic cells from ptc+/− mice (another SHH medulloblastoma model) [225].

The better outcome in WNT MB has been hypothesized to be due to the secretion of a soluble Wnt antagonist like Dickkopf 1 (DKK1) or the Wnt inhibitory factor 1 (WIF1) by tumor cells, which may reduce the Wnt/β-catenin pathway activity in neighboring endothelial cells, damaging the blood–brain barrier and making the tumor more susceptible to chemotherapy [226]. In addition, Manoranjan proposed that the inhibition of self-renewal pathways such as Bmi1 and Sox2 could explain the improved outcome in WNT medulloblastoma [215,227]. Bmi1 and Sox2 are hallmark master regulatory stem cell genes, essential for brain tumor initiating cell (BTIC) self-renewal [228,229,230]. A lesser expression of both Bmi1 and Sox2 is observed in WNT medulloblastoma BTICs compared to non-WNT cells. Ectopic Wnt activation in primary patient-derived medulloblastoma lines proved that the activation of the Wnt/β-catenin pathway in WNT3A-conditioned medium significantly reduced the self-renewal capacity along with the Sox2 and Bmi1 expression in Group 3 and Group 4 medulloblastoma lines.

Orthotopic injections of WNT medulloblastoma to smaller, less invasive tumors increased mouse survival rates with respect to Group 3 xenograft mice [215]. Meanwhile, animals with a Group 3 xenograft with ectopic β-catenin activation showed a notably decreased tumor burden and a higher overall survival (OS) compared to control mice [215]. Furthermore, the treatment of Group 3 and -4 xenograft with CHIR99021 (a GSK-3β inhibitor) improved survival by activating β-catenin, increasing *axin2* mRNA and downregulating *Sox2* and *Bmil*; L807mts, another GSK-3β inhibitor, can induce Wnt/β-catenin signaling in Group 3 and -4 medulloblastoma xenografts, decreasing tumor burden and increasing survival with respect to control xenografts [215].

Some authors suggested that the inhibition of stemness determinants could be a therapeutic strategy to transform malignant tumors into less aggressive cancers, like medulloblastoma [215,227]. Furthermore, it has been observed that β-catenin activation enhances radiosensitivity and decreases the invasive capacity of medulloblastoma cells [231], while a pharmacological activation of WNT by lithium reduced radioresistance in *TP53*-mutant medulloblastoma [232]. The poor survival of *TP53*-mutant medulloblastoma patients could be correlated to radiation resistance [233]. The constitutive activation of the Wnt/β-catenin pathway by lithium sensitized *TP53*-mutant medulloblastoma cells, and it could represent an attractive therapy for high-risk medulloblastomas [232].

In addition, Salaroli et al. showed that radiation triggers Wnt/β-catenin pathway activation with the ensuing cell cycle arrest and apoptotic cell death in medulloblastoma DAOY (*p53*-mutated) and D283MED (wild-type *p53*) cell lines; apoptosis and growth arrest in TP53-mutated medulloblastoma cells could be mediated by Wnt/β-catenin, bypassing the p53 pathway [234].

A dysregulated β-catenin signaling leads to an increased p53 transcriptional activity through ARF, which blocks the proteasomal degradation of p53 mediated by Mdm2 [235]. Not only β-catenin can affect the levels of p53, but p53 can in turn modulate the levels of β-catenin, enhancing its proteasomal degradation, possibly through CKI and GSK-3β, which are involved in β-catenin phosphorylation on key amino-terminal serine residues [236], since β-catenin is free to exert its functions in the absence of a functional p53 pathway. Interestingly, wild-type TP53 cells cannot downregulate mutant β-catenin [235,237].

These results suggest that β-catenin can modulate the expression of stem cell genes and induce apoptosis by p53-dependent and independent mechanisms in medulloblastoma cells.

### 4.6. Hepatocellular Cancer

Hepatocellular cancer is the fourth leading cause of cancer-related death; it ranks sixth in estimated incidence, estimating approximately 810,000 liver cancer-related deaths per year [238]. An increase up to 43% in mortality rates was reported for hepatic cancer in the USA in the 2000–2016 period. Several risk factors, including genetic mutations, long-term virus infection, and cirrhosis, have been described to predispose to hepatocellular cancer [239]. Therefore, discovering appropriate molecular targets and developing new therapeutic strategies is much needed to improve patient survival. Understanding what genetic and molecular lesions lead to liver cancer progression could provide fundamental insights for clinical applications.

Kim et al. demonstrated that the loss of the Hippo kinases Mst1/2 in hepatocytes induces the activation of Notch signaling and establishes a positive feedback loop with the Yes-associated protein/WW domain containing the transcription regulator 1 (YAP/TAZ), which rapidly results in hepatomegaly and hepatocellular cancer [240,241]. The activation of the Wnt/β-catenin signaling pathway blocks the positive feedback loop between Notch signaling and YAP/TAZ, preventing hepatocellular cancer. Mechanistically, the inactivation of Mst1/2 induces the activation of YAP/TAZ, which upregulates the expression of the Notch ligand Jag1 and triggers Notch signaling activity. The binding of Jag1 to Notch receptors results in the proteolytic cleavage of receptors by the γ-secretase complex and proteins of the ADAM family, to expose the Notch intracellular domain (NICD), which increases the activity of TAZ by blocking its binding to the E3 ubiquitin ligase β-TrCP; this induces a rapid tumor initiation and progression in hepatocytes with a Hippo signaling inactivation.

Wnt/β-catenin signaling suppressed the positive feedback loop between Notch and TAZ, by inducing the nuclear relocation of DP1 (the dimerization partner of E2F transcriptional factors), which inhibits Notch signaling by promoting NICD degradation. In addition, the inhibition of Notch signaling in vivo significantly reduced the YAP/TAZ activity, along with liver tumorigenesis [240,241]. Those authors suggested the existence of molecular interplay between the Hippo/Notch and Wnt/β-catenin signaling pathways to maintain liver size and suppress hepatocellular carcinoma [241]. It has been proposed that Disheveled physically interacts with the Notch carboxyl terminus end, blocking the Notch signaling [56]. In HepG2 hepatoma cells, pβ-catenin has been described to promote TAZ degradation by bridging TAZ to the β-TrCP complex [49]. About 30% of hepatocellular cancer cases show an inactivation of the tumor-suppressing proteins Mst1 and Mst2, and consequently, a high YAP/TAZ activity, indicating the contribution of Hippo in hepatocellular cancer [242].

LATS2 overexpression induces apoptotic cell death in SMMC-7721 and BeL-7404 liver cancer cell lines [243]. The apoptotic effect of LATS2 is correlated with an activation of the dynamic related protein-1 (DRP1)-related mitochondrial division via the Wnt/β-catenin pathway [243]. It was already known that the inhibition of Wnt/β-catenin signaling by DKK reduced the LATS2-mediated overexpression of DRP1, with the ensuing block in ROS generation and caspase-3 activity, while increasing cellular adenosine triphosphate (ATP) production [243]. LATS2 overexpression and the activation of DRP1-related mitochondrial division via Wnt/β-catenin could open promising strategies to treat hepatocellular cancer by inducing cell viability downregulation, mitochondrial dysfunction, energy depletion, and apoptosis activation on liver cancer cells [243]. An excessive mitochondrial division promotes the depolarization of mitochondrial membrane, triggering the opening of mitochondrial permeability transition pores (mPTP), the release of cyt c into the nucleus, and finally the activation of caspase-3 and -9, inducing apoptosis [244].

Qing-Qiang et al. demonstrated that trichostatin A (a deacetylase inhibitor) inhibits cell proliferation and induces cell cycle arrest and apoptosis on HepG2 hepatoma cells by decreasing the activity of histone deacetylases (HDAC1 and HDAC3) and activating β-catenin [245]. When chromatin is relaxed, β-catenin was proposed to bind TCF and chromatin-modifying factors, allowing the activity of the transcription complex and RNA polymerase to stimulate the transcription of downstream genes, including *Bax, Bcl-2, p2I,* and *p53,* thus decreasing cell survival and favoring apoptosis in HepG2 cells [245,246].

Overall, these results suggest that the Wnt/β-catenin pathway induces an antineoplastic effect by engaging in crosstalk in Hippo and Notch signaling, along with other pathways.

### 4.7. Hematologic Neoplasms

Over the years, the role of the Wnt/β-catenin pathway in the embryologic development of hematopoietic stem cells (HSCs), as well as in the pathogenesis and progression of cancer, has been studied. Various gene mutations in Wnt signaling molecules have been proved to induce a cytoplasmic or nuclear accumulation of β-catenin. Such mechanism has been observed in several neoplasm types, including hematologic ones [247]. Thus, different studies have centered on modifying the expression of β-catenin in hematologic models. Herein, we will focus on two of the most aggressive forms: Acute leukemia and multiple myeloma.

In the first place, Wnt/β-catenin activation was observed to induce apoptosis, as proved by β-catenin stabilization both in in vivo and in vitro models; mutant HSCs with deletion of exon 3 in the *Ctnnb-1* gene, which induced a higher expression of activated β-catenin, were found to downregulate *Bcl2* and upregulate *Casp3* with respect to controls [248]. In fact, an activation of the Wnt/β-catenin signaling pathway activated caspase-3 and -9, reducing the mitochondrial membrane potential and inducing apoptosis [248].

Histone acetylation and deacetylation play a major role in tumorigenesis. Acetylation is known to activate transcriptional activity, while deacetylation inhibits it [249]. Shao N. et al. reported an increased WNT/β-catenin activation when administering valproic acid (VPA) and suberoyl bis-hydroxamic acid (SBHA), both being HDACis, to T-cell acute lymphoblastic leukemia (T-ALL); both agents inhibited proliferation of Jurkat and Molt-4, T-ALL cell lines with a low β-catenin expression [250]. An arrest of the cell cycle in the G_2_/M phase, a lower number of cells in the S phase, and increased levels of p21^WAF1^, a key mediator in cell cycle arrest, were also observed. Meanwhile, an upregulation of β-catenin induced apoptosis [250].

A dose-dependent loss of cell viability was observed with Wnt agonists in a monocytic leukemia model using the RAW 264.7 and J774.1 murine cell lines. The binding of Wnt agonists to their receptors led to dysfunctional activity of GSK-3β, along with β-catenin stabilization and nuclear accumulation [251].

In a novel approach, RNA interference has been used to silence genes in oncology [252]. Particularly, multiple myeloma cell models have been infected with lentivirus vectors coding for a small transfer (si) RNA specific for β-catenin. Infecting RPMI-8826 multiple myeloma cells with lentivirus strains that express siRNA (8826-sc) or three β-catenin siRNAs (8826-siA, siB, and siC) led to a decrease in protein expression and cell viability [247]. Infected cells also underwent autophagy, as demonstrated by the induction of LC3-I, which cleaved LC3-II, resulting in increased LC3-II and decreased LC-I levels, while higher Beclin-1 levels were also observed [247].

On the other hand, higher apoptosis rates were observed in multiple myeloma cells with a silenced β-catenin pathway, increasing the expression of phosphorylated p53, active caspase-3, and Bax, while the expression of Bcl-2 was decreased, suggesting a mitochondrial involvement [247]. Intervening in the regulation of the β-catenin pathway in treatment-resistant myeloma multiple cells is a promising therapeutic approach. LiCl has showed a cytotoxic effect on the RPMI-8226 and U266 multiple myeloma cell lines, while it induced cell cycle arrest, increased the fraction of cells entering in the G_2_ phase, and decreased the expression of cyclin D1 [253]. LiCl inhibits cell viability in the RPMI-8226/BTZ100 cell line, which is resistant to bortezomib (BTZ) [253]. A deeper study of the action mechanism of LiCl showed it increases the levels of pGSK-3β, inhibiting the activity of GSK-3β; as a result, β-catenin is not phosphorylated, being translocated into the nucleus instead [253].

### 4.8. Other Neoplasms

Mst1 overexpression causes apoptosis by increasing ROS generation, the levels of the Bax proapoptotic protein, and the activity of caspase-9, while decreasing the antioxidant activity of SOD, GSH, and GPX, as well as the levels of the antiapoptotic protein Bcl-2 in the squamous cell carcinoma of the head and neck (SCCHN) Cal27 cell line and in Tu686 cells; the mechanism is thought to involve the regulation of mitochondrial fission via the β-catenin/DRP1 pathways [254]. An inhibition of the Wnt/β-catenin pathway by DKK1 was already reported to lower Mst1-mediated DRP1 upregulation, and therefore apoptosis, in Cal27 and Tu686 cells [254].

Mst1 overexpression has also been reported to decrease cell viability and induce apoptotic cell death in the human non-small cell lung cancer (NSCLC) A549 line both in vitro and in vivo, by promoting the cytosolic relocation and Ser^127^ phosphorylation of YAP, as well as inducing a decrease in amphiregulin, the connective tissue growth factor, and *Survivin* mRNA [255]. Mst1 also induced mitochondrial apoptosis, inhibiting cell migration in the A549 cell line by increasing ROS generation, reducing mitochondrial potential, overexpressing proapoptotic proteins like Bad, caspase-3, and caspase-9, while downregulating the antiapoptotic proteins c-IAP and Bcl-2, and promoting cyt c translocation into the nucleus by activating ROCK1 and decreasing F-actin stabilization [256]. In addition, it has been reported that ROCK1 prevents the epithelial–mesenchymal transition in patients with lung neoplasms [257].

On the other hand, a lower β-catenin expression correlated with a poorer prognosis [258], a more advanced tumor stage, and shorter patient survival in non-small cell lung cancer [259,260]. You et al. reported that that β-catenin-overexpressing H460 lung cells were more susceptive to TRAIL, favoring apoptosis via death receptor by promoting the relocation of DR4 and DR5 to the plasmatic membrane [206].

The tumor suppressor candidate 3 (TUSC3) inhibited cell growth and induced apoptosis in A549 cells. TUSC3 also promoted increased levels of LC3-II and Beclin-1, favoring autophagosome formation, while lowering the phosphorylation (inactivation) of β-catenin and promoting its nuclear translocation [261]. The loss of TUSC3 in A549 cells inhibits autophagy by increasing the SQSTM1/p62 expression [262].

Other studies have described a nuclear induction of β-catenin in human pancreatic cancer cells by SAHA, which resulted in an inhibition of cell proliferation and in apoptosis induction [263]. The upregulation of β-catenin and E-cadherin in MiaPaca2 pancreatic and HS785T breast cancer cells induces apoptosis, inhibiting cell migration and invasion. It was suggested that the formation of an E-cadherin/β-catenin complex is required for apoptosis induction, which is independent of β-catenin/TCF transactivation [264]. A lower expression of the E-cadherin/β-catenin complex was found to correlate with a poorer prognosis and lower survival in esophageal adenocarcinoma and gastric cancer [265,266].

Interestingly, Ursolic acid exerts its antineoplastic effect on SK-OV-3 ovarian cancer cells by inducing β-catenin stabilization and promoting the phosphorylation (inactivation) of GSK-3β [267]. Treating embryonal rhabdomyosarcoma (ERMS)-delivery zebrafish with BIO induced the stabilization of the Wnt/β-catenin pathway, decreasing ERMS proliferation and the number of self-renewing tumor generating cells [8]. Additionally, treating human alveolar rhabdomyosarcoma ARMS cell lines with WNT 3A induced the stabilization of β-catenin and its nuclear location and activation, leading to a marked decrease in proliferation levels and in the induction of the myogenic differentiation markers myf5, myogenin, and MyoD [268].

It was reported that LY20314 (a GSK-3β inhibitor) inhibits cell proliferation in human neuroblastoma cell lines while increasing β-catenin levels [269]. Duffy et al. reported that a combined treatment with GSK-3β inhibitors like LiCl or BIO-acetoxime and a Wnt agonist induced death on neuroblastoma cells, downregulating c-myc and *MYCN* mRNA expression, regulating the p53 and Wnt/β-catenin pathways [270]. The *MYCN* oncogene is amplified in 25% of neuroblastomas, and it has been linked to therapeutic resistance [271]. *MYCN* overexpression in neural crest progenitor cells leads to neuroblastoma formation [272]. It has been demonstrated that both β-catenin overexpression and downregulation promote an antineoplastic effect on *MYC*-amplified neuroblastoma. Treatment with ICG-001 (a canonical Wnt pathway inhibitor) and the Wnt agonist 1 (a canonical Wnt pathway activator) significantly impaired cell viability in IMR32 neuroblastoma cells [17]. Wnt agonist 1 downregulated *MYC* mRNA levels and decreased cell viability by inducing apoptosis, while ICG-001 increased *MYCN* transcripts and reduced cell viability and proliferation.

These results suggest that altering β-catenin thresholds in a cancer cell, either below or above normal levels, will be detrimental to cell survival. MYCN has also been suggested to block the function of Wnt/β-catenin signaling by promoting the binding of MYCN to β-catenin, thereby stopping its transcriptional activity [17]. On the other hand, the normal genetic regulation of β-catenin can be altered by directing MYCN to β-catenin target genes [17].

It has been proposed that TGF-β induces apoptosis in prostate cancer cells through Smad 7, which interacts with TAK1 and MKK3, leading to the activation of p38 and the ensuing activation of AKT, the inactivation of GSK 3β, and the stabilization of β-catenin [53,273]. Furthermore, TGF-β has been demonstrated to induce apoptosis in prostate cancer cells through the association between Smad7, β-catenin, and TCF, leading to the expression of c-myc [274]. On the other hand, Smad7 binds Axin, promoting the inactivation of the degradation complex and the stabilization of β-catenin [53].

## 5. Conclusions

The works herein reviewed suggest that, under certain circumstances, it is possible to induce apoptosis in cancer cells by activating the Wnt/β-catenin signaling pathway and/or receptors with tyrosine kinase activity that leads to β-catenin stabilization, either by deactivating the degradation complex, α-catenin, or the Notch signaling pathway, or by activating the Hippo pathway, which in turn inactivates the YAP/TAZ transcriptional factors.

**Figure 4:**
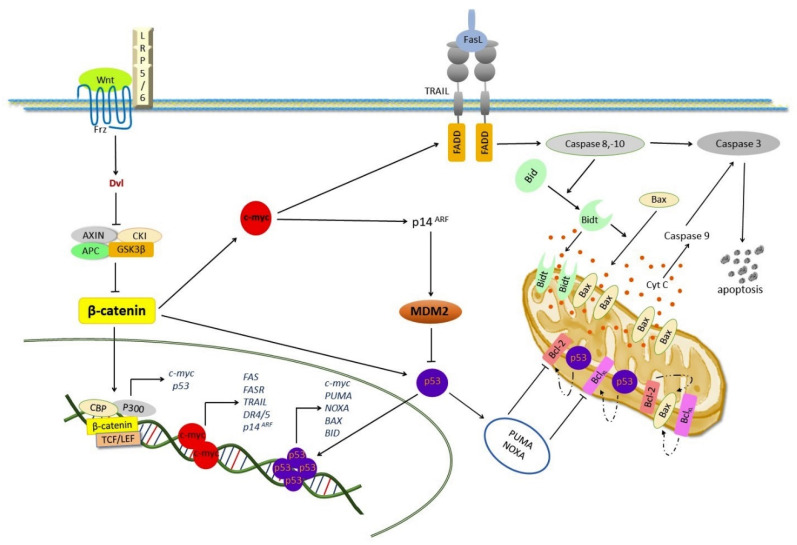
Suggested pathway initiated by β-catenin to promote the induction of apoptotic cell death on cancer cells. β-catenin can be stabilized by the Wnt/β-catenin pathway and/or the EGF and TGF-β signaling pathways. β-catenin can bind and activate TCF4/LEF, which may activate the transcription of p53 and c-myc. C-myc can increase the genic expression of p14^ARF^, fas, Trail, fasR, and DR4/5. FasR and DR4/5 activate the apoptotic extrinsic pathway, which is initiated by the binding of their respective ligands. This leads to the autoactivation of caspases-8 and -10, which in turn promote the catalytic activation of the effector caspase-3. Another target of caspase-8 is the pro-apoptotic protein Bid, which is hydrolyzed to tBid, inducing Bax oligomerization and mitochondrial depolarization with release of cyt c. Along with the activation of caspase-9, these events amplify the apoptotic pathway. On the other hand, p14^ARF^ inhibits mdm2, which induces the ubiquitination and ensuing degradation of p53. p53 can induce apoptosis via transactivation of pro-apoptotic genes such as *Noxa, Puma, Bax,* and *Bid*, which inhibit the Bcl-2 and Bcl_XL_ anti-apoptotic proteins. p53 also acts by directly inhibiting Bcl_XL_ and Bcl-2 in the mitochondria, inducing the permeabilization of the mitochondrial outer membrane, with the ensuing release of cyt c y the activation of apoptosis intrinsic pathway. Furthermore, p53 induces c-myc genic expression. Continue arrows (↓) indicate activation, arrows with (⊥) indicate inhibition.

All these events result in a release and accumulation of β-catenin, causing its nuclear translocation and its transactivating capacity. Among other mechanisms, the Wnt/β-catenin pathway can induce apoptosis by downregulating antiapoptotic proteins and inducing the transcription of p53 and c-myc, which in turn can induce the transcription of proapoptotic proteins (Figure 4).

However, the effect of the Wnt/β-catenin signaling pathway in cancer cells is extremely complex and context-dependent. It can be involved in the regulation of cell growth, proliferation, and death. For years, a dysregulation of the Wnt/β-catenin pathway was regarded as contributing to cancer invasion and metastasis, modulating the tumor microenvironment, and promoting resistance to treatment. Nevertheless, further studies indicated that this initial picture was an oversimplification. Instead, the Wnt pathway, either acting by a β-catenin-dependent or an independent mechanism, can inhibit or promote cancer progression in a stage- and cancer-type-specific manner.

On the other hand, it has been proposed that the Wnt/β-catenin signaling and its crosstalk with other signaling pathways, including Hippo, DRP1, Notch, EGFR/RAS/RAF/MEK/ERK, LKB/AMPK, Rb, c-myc, p53, and FOXO, plays a key role in the regulation of cell growth, proliferation, and death. The study of the crosstalk between Wnt/β-catenin signaling and other well-characterized signaling pathways will deepen our understanding of the cell signaling network. In time, it may help us to discover valuable therapeutic targets to treat cancer.

## Data Availability

Data is contained within the article.

## Abbreviations

5′-AMP-activated protein kinase	AMPK
Adenomatous polyposis coli	APC
Adenosine monophosphate	AMP
Adenosine triphosphate	ATP
Apoptotic protease-activating factor 1	apaf-1
Apoptosis inhibitor	IAP
BCL2 Associated X	BAX
B-cell lymphoma 2/B-cell lymphoma 9	Bcl-2 / Bcl-9
B-cell lymphoma-extra-large	*Bcl*-*xL*
Bcl-2-like protein 11	BIM
BH3-interacting domain death agonist	BID
Brain tumor initiating cell	BTIC
Central nervous system	CNS
Chaperone-mediated autophagy	CMA
c-Jun N-terminal kinase	*JNK*
Colony-stimulating factor-1	CSF1R
C vacuolar protein	C-VPS
Cytochrome c	cyt c
Dapper homolog 2	DACT2
Dishevelled	Dvl
Death domain	DD
Death effector domain	DED
Death-inducing signaling complex	DISC
Deoxyribonucleic acid	DNA
Docosahexaenoic acid	DHA
Element-binding protein	CBP
Epidermal growth factor receptor	EGFR
Extracellular-signal-regulated kinase	ERK
Fas-associated death domain-containing	FADD
Forkhead box class O	FOXO
Glycogen synthase kinase 3β	GSK-3β
Glioblastoma multiforme	GBM
Glioma Stem Cells	GSC
GSK-3β binding protein	GBP
Guanosine triphosphatases	GTPases
High-temperature requirement A2	Omi/HtrA2
Histone deacetylase	HDAC
Liver kinase B1	LKB1
Low-density lipoprotein receptor-related proteins 5/6	LRP5/6
Mammalian sterile-20 kinase 1 and 2	MST1/2
Matrix metallopeptidases	MMP
Methylguanine-O^6^-methyltransferase	MGMT
Mitochondrial outer membrane permeabilization	MOMP
Mitogen-activated protein kinase	MAPK
Mammalian target of rapamycin	mTOR
Myeloid cell leukemia 1	Mcl-1
Nuclear factor kappa-light-chain-enhancer of activated B cells	NF-κB
Overall survival	OS
Planar cell polarity	PCP
Phorbol-12-myristate-13-acetate-induced protein 1	NOXA (PMAIP1)
Protein endoplasmic reticulum kinase	PERK
p53-induced death-domain-containing protein	PIDD
p53 upregulated modulator of apoptosis	PUMA
Pyruvate kinase M2	PKM2
RAS-related C3 botulinum toxin substrate 1	RAC1
Reactive oxygen species	ROS
Rho-associated kinase	ROCK
Salvadoran homoloid 1	SAV1
Second Mitochondria-derived Activator of Caspases	*SMAC*
Sequestosome 1	SQSTM1
Tafazzin	TAZ
Target of rapamycin complex 1	TORC1
Truncated Bid	tBID
T cell factor/lymphocyte enhancer	Tcf/Lef
Temozolomide	TMZ
Transcription factor EB	TFEB
Tumor Necrosis Factor receptors	TNFR
Tumor necrosis factor-related apoptosis-inducing ligand	TRAIL
Tumor necrosis factor-related apoptosis-inducing receptor	TRAIR
yes –associated protein	YAP
